# Spatial organization and dynamics of genome replication: from forks to foci

**DOI:** 10.1093/nar/gkag550

**Published:** 2026-06-08

**Authors:** Sunil K Pradhan, Maria Arroyo, Maruthi K Pabba, Heinrich Leonhardt, M Cristina Cardoso

**Affiliations:** Cell Biology and Epigenetics, Department of Biology, Technical University of Darmstadt, 64287 Darmstadt, Germany; Department of Medical Biochemistry and Biophysics, Karolinska Institutet, 17177 Stockholm, Sweden; Science for Life Laboratory, 17165 Solna, Sweden; Cell Biology and Epigenetics, Department of Biology, Technical University of Darmstadt, 64287 Darmstadt, Germany; Cell Biology and Epigenetics, Department of Biology, Technical University of Darmstadt, 64287 Darmstadt, Germany; Human Biology and BioImaging, Faculty of Biology, LMU Munich, 81377 Munich, Germany; Cell Biology and Epigenetics, Department of Biology, Technical University of Darmstadt, 64287 Darmstadt, Germany

## Abstract

Genome replication is without doubt the most complex and critical activity of all living cells. Any error may not only affect the cell itself but may lead to disease and, thus, kill the entire organism or be transmitted to subsequent generations via the germline. The task of replicating the genome “once and only once” becomes increasingly challenging with larger genomes and requires efficiency paired with coordination within the cell nucleus. While biochemical studies using frog egg extracts and genetic studies using bacteria and yeast have identified the basic machinery, it was the combination with cellular and molecular studies that yielded a comprehensive view of genome replication. Here, we focus on DNA replication in mammals as they pose a challenge in terms of genome size (and age) and total number of cell division cycles per lifetime, coupled with a variety of pathologies. We discuss how studies at the cellular level provided a framework for understanding the progression of DNA replication throughout the S phase. We highlight how pulse labeling and time lapse studies in combination with genome sequencing technologies are providing a comprehensive view of how large genomes are efficiently and precisely replicated every day in trillions of cells.

## Graphical Abstract

**Graphical Abstract:**
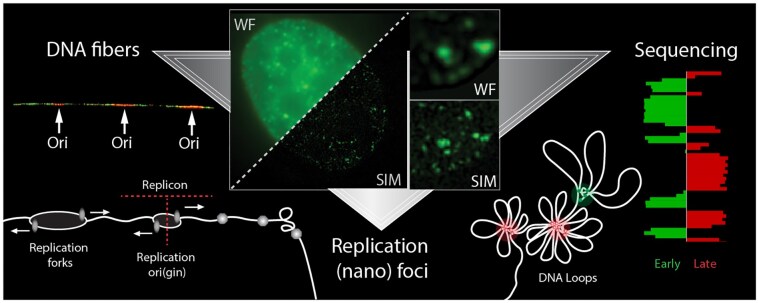

## Introduction

The enormous challenge of DNA replication in a human body is best described with the sheer size of DNA synthesized per day. Taking into account that ~2% of all cells in a human body (total ~28–36 trillion cells) [1] corresponding to a little <1 trillion cells (including mostly epithelial and hematopoietic cells) replicate the genome per day, this entails the synthesis of 1 trillion times 2 m of DNA (estimate of the length of the human genome). This means two trillion meters of DNA are synthesized per day in the body, which is roughly equivalent to 10 times the distance from earth to sun (assuming 150 million km distance). Here, we will discuss relevant concepts as well as the technologies to study genome replication from the DNA fiber to replication foci (RFi) in cells and point out strengths and weaknesses of current experimental methods and findings.

The biological framework underlying genome replication involves a conserved set of molecular mechanisms encompassing origin licensing by the origin recognition complex (ORC) and MCM helicase loading, origin activation through the concerted action of kinases (CDK and DDK) and additional firing factors, processive fork elongation by the helicase and associated replicative polymerases, and termination. Cell cycle checkpoints coordinate these steps to ensure complete and accurate genome duplication. While these molecular mechanisms have been extensively reviewed elsewhere [2–5], understanding how they operate within the spatial and temporal context of the cell nucleus remains a central challenge. In this review, we focus on how cellular and genomic approaches have revealed the higher-order organization of DNA replication. We start with the first visualization of replication forks on stretched DNA fibers and the detection of RFi in intact cells up to the genome-wide mapping of replication origins and timing domains and how these scales relate to chromatin structure and nuclear architecture.

### From the DNA double helix to replication forks

The proposal of the DNA double helix model structure by Watson and Crick in 1953 [6] and the experimental confirmation of the postulated semiconservative DNA replication by Meselson and Stahl in 1958 [7] immediately provided a conceptual framework for understanding how genetic information could be duplicated. However, it was the advent of DNA fiber techniques in the 1960s that paved the way to directly visualize and analyze replication dynamics at the single-molecule level.

The pioneering work of Huberman and Riggs in the late 1960s [8] combining pulse labeling with tritiated thymidine followed by genomic DNA fiber autoradiography allowed the observation and quantitation of replication origins and bidirectional replication fork movement as well as determination of fork rate in mammalian chromosomes. It is worth noting that these early fiber autoradiography experiments required exposure times of 4–8 months! The theoretical foundation provided by the double helix model and the empirical power of DNA fiber analysis yielded insights into DNA replication, which are applicable to all life forms. In Table 1, landmark studies using DNA replication and DNA fiber analysis are compiled from the early 1960s to the 2020s.

**Table 1.** History of DNA fiber analyses in DNA replication studies

Year	Landmark	Author
1962/1966	The first replication-labeled DNA fibers in *Escherichia coli* using autoradiography	Cairns [9]
1968	Visualization of replication forks and fork speed measurement in mammalian cells	Huberman and Riggs [8, 10]
1974	Electron microscopy technique, multiple forks, and bidirectionality in *Drosophila*	Kriegstein and Hogness [11]
1977	Relation between numbers of replicon and total S-phase timing, replicon clustering concept	Yurov and Liapunova [12]
1993	FISH on DNA fibers	Parra and Windle [13]
1994	DNA combing (relation between stretching DNA length in kb)	Bensimon *et al*. [14]
1995	DNA fiber FISH of large genes in human DNA	Florijn *et al*. [15]
1997	Molecular combing of human genome at high resolution	[16]
2000	Replication fork density increase in *Xenopus* egg extracts	Herrick *et al*. [17]
2002	Origin mapping at rDNA loci using FISH on DNA fiber analysis in yeast	Pasero *et al*. [18]
2005	Role of histone modifications on DNA replication using DNA combing combined with FISH in mammalian cells	Takebayashi *et al*. [19]
2005	Mapping origins at immunoglobulin heavy chain locus using FISH on DNA fiber analysis in B cells	Norio *et al*. [20]
2012	Telomere FISH and DNA replication combing	Drosopoulos *et al*. [21]
2021	Genome-wide optical replication mapping in human cells	Wang *et al*. [22]
2022	Open source software for high throughput DNA fiber analysis	Li *et al*. [23]

FISH: Fluorescence *in situ* hybridization

**Figure 1.**
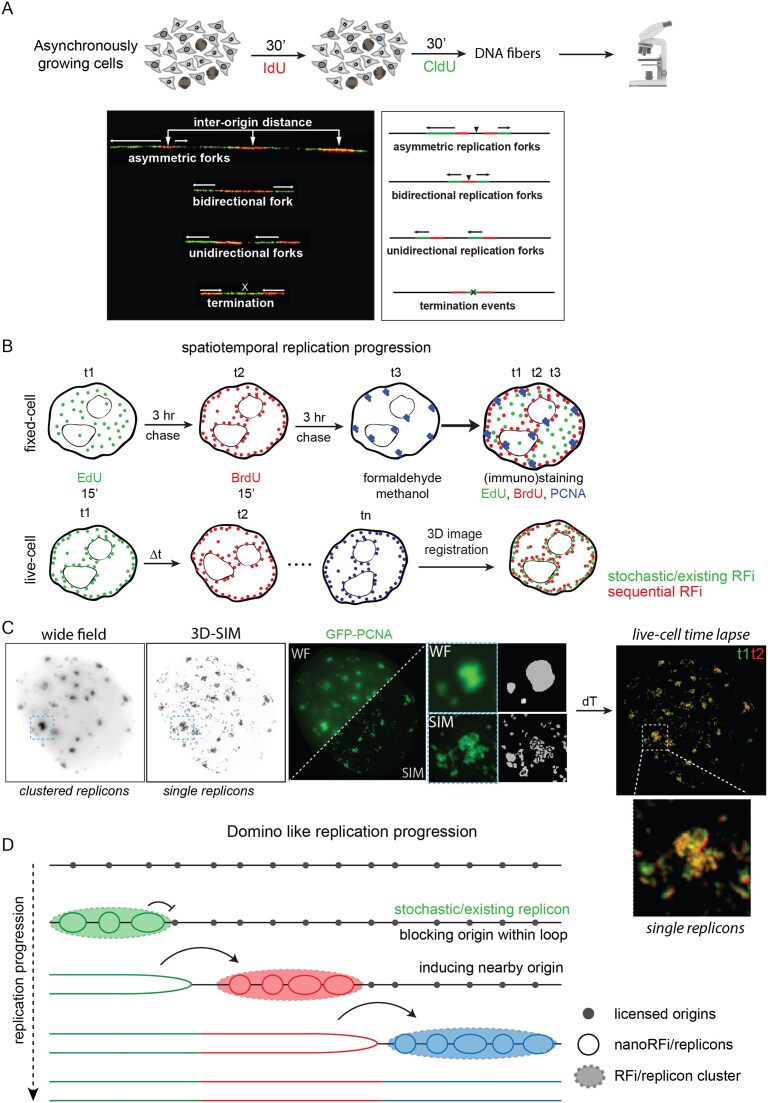
Dissecting the spatiotemporal genome replication program. (**A**) Schematic representation of the double nucleoside pulse labeling approach followed by genomic DNA isolation and spreading on a glass slide, detection of incorporated nucleotides, and imaging. Single DNA fiber images and graphical interpretation of inter-origin distance measurements, bidirectional and unidirectional forks, and symmetrical and asymmetrical forks as well as fork termination events. Several additional DNA replication parameters (e.g. replication fork speed) can be derived from such analysis. (**B**) Schematic representation of the experimental workflow combining fixed-cell and live-cell microscopy to investigate the spatiotemporal progression of genome replication at different time (Δ*t* = time course) and space resolutions. In the fixed-cell microscopic analysis, the cells are incubated with nucleoside analogs for short labeling pulses followed by long chase times to resolve spatial patterns within sub-stages of the S phase. These patterns are visualized by detecting the incorporation of the nucleoside analog in the genomic DNA during DNA synthesis and the replication machinery components (e.g. the polymerase clamp PCNA). Live-cell microscopy tracks the dynamics of the replisome machinery (e.g. GFP-PCNA) over time. To correct for cellular motion and deformation, image registration is applied, allowing to distinguish the pre-existing from the newly activated RFi. This time overlay analysis revealed a sequential, domino-like genome replication progression, whereby existing RFi are spatially associated with the subsequent firing of adjacent RFi. (**C**) Correlative microscopy of wide-field and superresolution followed by (nano)RFi segmentation revealed multiple nanoRFi (aka, replicons) within one RFi corresponding to a cluster of adjacent replicons that fire in a coordinated manner. Live-cell superresolution microscopy revealed the dynamics of individual replicons inside the clusters (RFi). (**D**) Illustration describing the domino model of replication progression: activated RFi (cluster of adjacent replicons) block the activation of licensed origins within an average DNA loop distance, and progression of replication at these sites eventually induces the activation of nearby (cluster of) origins.

The DNA fiber replication technique and corresponding data analysis dramatically evolved with the development of antibodies to halogenated nucleoside analogs including 5-bromo-, 5-iodo-, or 5-chloro-deoxyuridine in the 1980s [24]. The latter allow the detection of newly synthesized DNA using fluorescence detection as opposed to the time-consuming autoradiography. Altogether, using such techniques, several of the most important concepts in DNA replication came to be, including the determination of replication origins and the estimates of their numbers; the measurement of origin distances within the DNA fiber (aka, indirectly, replicon size); the determination of replication fork rate and bidirectionality as well as replication fork asymmetry and/or unidirectionality; the determination of fork stalling as well as fork termination events; and several other measurements in the linear DNA fiber (Fig. 1A). Importantly, the combination of replication labeling with the tracking of the DNA replication machinery made it possible to relate replication forks to the spatiotemporal organization of genome replication (nano)RFi within the nucleus as outlined in Fig. 1B–D and discussed in more detail in subsequent sections.

**Figure 2.**
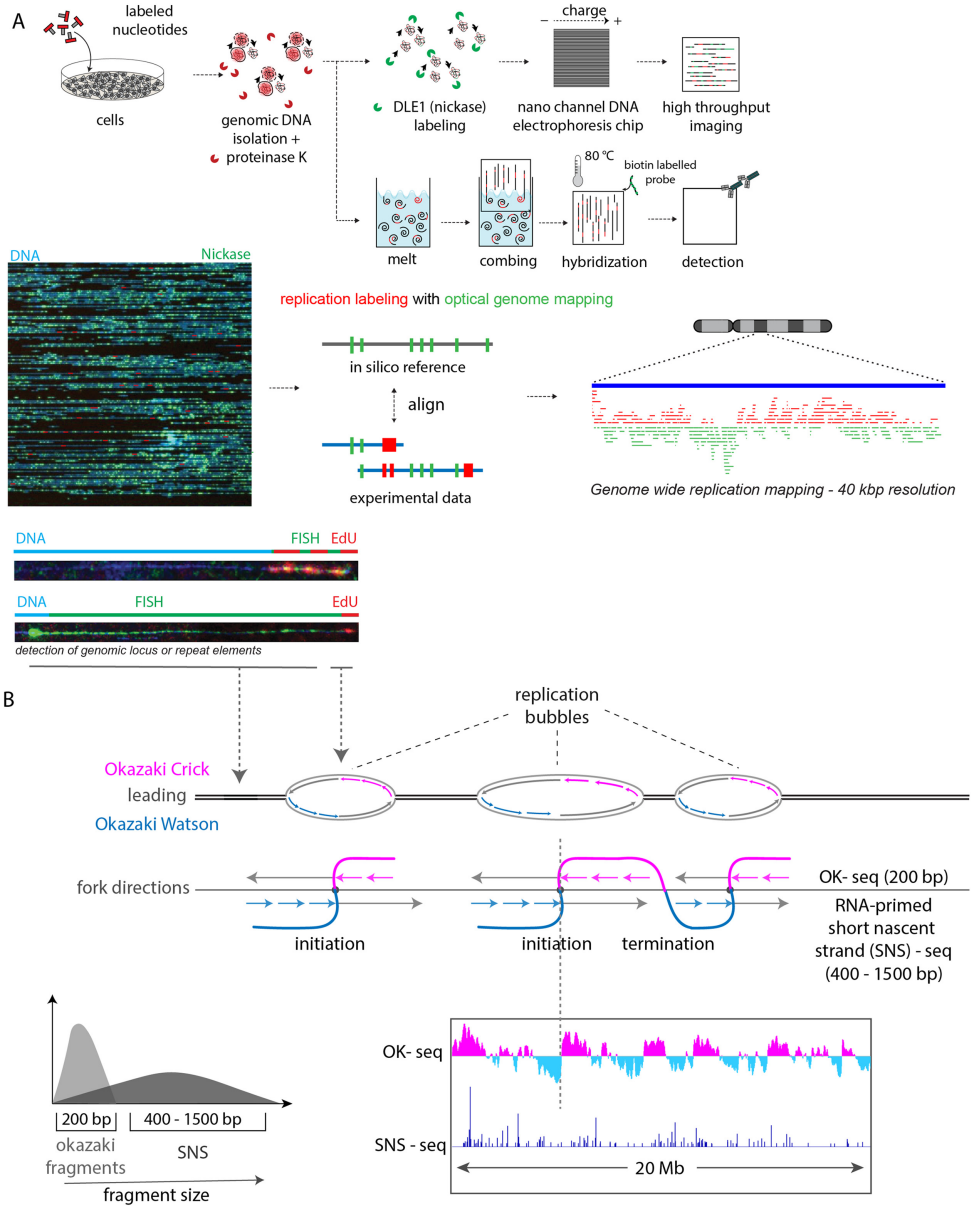
Dissecting genome-wide DNA replication initiation. (**A**) Evolution of DNA fiber analysis to determine replication origins across the genome. DNA fiber analysis using pulse-chase labeling enables visualization of distinct replication patterns, including bidirectional, asymmetric, and unidirectional forks and termination events at the single fiber level. Combined with FISH, this technique reveals replication dynamics at specific genomic locations. For genome-wide analysis, multiplexed approaches combining optical genome mapping with optical replication mapping permit the identification of replication origins, fork directionality, and termination sites across the entire genome. (**B**) Various next-generation DNA sequencing approaches for mapping the replication origins have yielded the genome-wide distribution of origins and their features (reviewed in Hu *et al*. and Hyrien *et al*. [2, 3]). The RNA-primed short nascent strands (SNS) technique takes advantage of the newly initiated strands of DNA, typically ranging in size from 0.4–2 kb, enriched around replication origins. Okazaki fragment sequencing (OK-seq) leverages the fact that replication forks produce Okazaki fragments (∼200 bp) asymmetrically on the lagging strands. The orientation and density of these fragments allow to infer replication initiation zones and fork direction. Example datasets show the initiation and termination zones inferred from OK-seq, while SNS-seq reveals the initiation zones along the genome. The OK-seq datasets were adapted from GEO: GSM3290342 [28, 29] and SNS-seq from GEO: GSE68347 [28, 29].

The major drawbacks of the DNA fiber technique have been the lack of DNA sequence information, the difficulty in obtaining intact long DNA fibers, and the low throughput and technical challenges inherent to all single-molecule methodologies. To obtain sequence information, two strategies have been devised. One approach is based on concomitant hybridization of DNA fibers with DNA probes for specific genomic regions (FISH, Fig. 2A) (see Table 1 for references). For single-copy genes, the usage of this approach is, however, like finding a needle in a haystack. Hence, this fiber–FISH combination approach has been mostly successful for repeat DNA elements (Table 1). The second approach relies on enzymatic deposition of fluorophores in a sequence-specific manner along the genome (Fig. 2A). This is performed together with the detection of the modified nucleotides incorporated during DNA replication and is therefore termed optical replication mapping [25, 22]. To increase the throughput and avoid the mixing of DNA fibers on the slide, two main approaches were developed. One approach is based on the silanization of the slides and the homogeneous stretching of the DNA fibers on the slide parallel to one another. This technique was developed in the 1990s and termed DNA combing [14, 17] (Fig. 2A). DNA combing has contributed significantly to our understanding of DNA replication in mammalian cells by allowing, at a higher throughput the direct visualization of newly synthesized DNA through the sequential pulse-labeling with nucleoside analogs, as described above for the DNA fiber spreading technique (Fig. 1A). While it provided unprecedented insights into replication dynamics, it came with significant limitations including laborious sample preparation, potential DNA breakage during stretching, and the challenge of mapping observed events to specific genomic locations. The integration of FISH with DNA fiber analysis provided some degree of sequence mapping, though this approach remained restricted to studying specific genomic regions. More recently, a second approach to increase the throughput was developed in which the genomic DNA fibers are spread along nano channels [26]. This was followed by a study combining enzymatic deposition of fluorophores in a sequence-specific manner along the genome with detection of modified nucleotides incorporated during DNA replication, with the genomic DNA fibers spread along nanochannels [27]. This technique allows a very high throughput and is also used in optical replication mapping. The development of optical replication mapping has addressed several limitations of the traditional fiber techniques by enabling genome-wide analysis of replication dynamics with sequence information. With this technique, one can simultaneously track thousands of replication events and map them to their genomic locations albeit the sequence resolution is not very high (Fig. 2A). However, optical replication mapping also presents its own challenges, including the requirement for specialized and expensive equipment, complex data analysis pipelines, and potential biases in DNA fragment size and coverage. Despite these limitations, the complementary use of traditional DNA fiber analysis and modern optical replication mapping techniques has significantly advanced our understanding of how replication programs are established and maintained in normal cells, and how they become dysregulated in disease states. Moving forward, integrating these approaches with other genomic methods (including high-throughput genome sequencing approaches, Fig. 2B) while addressing their technical limitations will be crucial for fully understanding the complexity of mammalian DNA replication. Most relevant in this context is the development of new computational analysis methods to extract relevant DNA replication parameters (origin mapping, replication fork speed, inter-origin distance, etc.). Although DNA fiber-based methods have been and continue to be very important in DNA replication studies, they provide a one-dimensional view of this DNA metabolic process. Furthermore, conceptually related to fiber-based approaches, nanopore sequencing has recently emerged as a powerful tool to study DNA replication at the single-molecule level with nucleotide resolution. These studies are discussed in more detail in the genome-wide replication mapping section below. Collectively, fiber-based studies established that mammalian chromosomes replicate from thousands of origins, that these origins fire in a coordinated rather than random manner, and that replicons cluster in groups that are activated together in time. Yet by their nature, fiber approaches capture replication as a linear, one-dimensional process and cannot address how these origin clusters are positioned and coordinated within the three-dimensional space of the nucleus, nor what determines their firing order throughout the S phase. These questions could only be answered by observing replication directly in the intact cell.

### From the DNA double helix to replication foci

Initially described many decades ago with the use of radioactively labeled thymidine, the concept that the mammalian genome replicates in a spatiotemporally ordered fashion, with genomic regions replicating earlier and others later in the S phase [30], highlights two critical aspects of eukaryotic DNA replication. First, not all replication origins are activated simultaneously and second, origins that do fire together are not evenly distributed across the genome [31]. These characteristics generate distinct replication patterns that follow a spatiotemporally conserved progression throughout the S phase. As mentioned above, such spatial replication patterns are now commonly visualized using fluorescence microscopy of fixed cells by detecting nucleoside analogs, such as BrdU, with specific monoclonal antibodies [24] and the more recently developed click chemistry EdU detection [32] (Fig. 1B). Over the years, several methodologies to deliver labeled dNTP nucleotides into cells have been used, including transient detergent permeabilization [33–36] or mechanical disruption of the plasma membrane [37, 38], microinjection [39–42], electroporation [22], [43–46], and cell-penetrating peptide-based techniques [47, 48]. These methods extended our ability to analyze DNA synthesis sites to living cells. Moreover, detection of replication machinery components either using antibodies or fluorescent protein fusions further expanded the possibilities of labeling and quantifying replication sites in fixed and living cells. These techniques are most often used in higher eukaryotic systems in part due to their large nuclei and the relative easiness to perform antibody detection of nucleosides and, in addition, replication machinery proteins. Table 2 highlights studies on DNA RFi in mammalian cells.

**Table 2.** History of DNA RFi studies

Year	Landmark	Author
1974	Newly synthesized DNA visualized by electron microscope autoradiography	Fakan and Hancock [49]
1982	Monoclonal antibody to 5-bromo- and 5-iododeoxyuridine (BrdU and IdU) for detection of DNA synthesis *in situ*	Gratzner [24]
1986	Detection of RFi in mammalian nuclei using fluorescence microscopy	Nakamura *et al*. [50]
1989	Distinct spatial patterns of RFi in S phase	Nakayasu and Berezney [33]
1992	Spatial patterns of RFi and co-detection with ɑ-satellite DNA	O’Keefe *et al*. [51]
1992	Progression of DNA synthesis using electron microscopy	Rizzoli *et al*. [52]
1993/1994	Concept of replication factories using electron microscopy	Hozak *et al*. (1993); Hozak *et al*. (1994) [34, 36]
1997	RFi in one-cell and two-cell mouse embryos	Ferreira and Carmo-Fonseca [53]
2000	Dynamics of RFi in living cells	Leonhardt *et al*. [54]
2002/2004/2005/2010	DNA polymerase clamp remains stable at active replication sites, with new assembly at nearby origin clusters: model of a domino-like replication progression	Sporbert *et al*. [55]Sadoni *et al*. [42]Sporbert *et al*. [56]Maya-Mendoza *et al*. [57]
2008	Development of 5-ethynyl-2′-deoxyuridine (EdU) click chemistry method for detection of DNA synthesis	Salic and Mitchison [32]
2009	Measurement of replication structures at nanometer scale	Baddeley *et al*. [58]
2016/2020	Determination that nanoRFi in mammalian cells correspond to individual replicons that cluster forming RFi	Chagin *et al*.; Xiang *et al*.; Rausch *et al*. [59–61]
2020	Effect of CTCF on RFi spatial distribution	Su *et al*. [62]
2020/2024	Mapping RFi to chromatin compaction classes at different microscopical resolution	Miron *et al*.; Pradhan *et al*. [63, 64]

Studies on genome replication in single-cell eukaryotes like yeast revealed well-defined replication origins that fired stochastically yet followed a generally conserved replication timing [65–67]. Higher eukaryotes including mammalian cells, despite having not defined origins, maintain a conserved replication timing, suggesting that ordered origin firing, rather than the origins themselves, plays a more significant role in faithful genome duplication [68, 69]. Accordingly, replication timing in mammalian cells detected with consecutive nucleotide pulses in fixed cells as well as in living cells by timelapse analysis of labeled replication machinery components (for methodology see Reinhart *et al*. [70]), depicted distinct spatial patterns of RFi, emerging progressively as the cell advanced through the S phase, and characterized into early, mid, or late sub S-phase stages (Fig. 1B) (reviewed in Chagin *et al*. [71]). To address which genomic regions replicated earlier or later in S phase, insights from FISH in cells or hybridization of target sequence on blots with nascent DNA demonstrated that specific chromosome regions are replicated at defined times within the S phase rather than randomly [51, 53, 67, 72, 73]). To elucidate the types of chromatin replicating earlier and late during S phase, replication labeling was employed in combination with markers for different chromatin types, including euchromatin, facultative, and constitutive heterochromatin including the inactive X chromosome in female cells. From these types of analyses, it was established that genome replication progression follows broadly genome compaction, with euchromatic regions replicating preferentially early and heterochromatin later (reviewed in Casas-Delucchi *et al*. [74]. These studies included diploid and tumor cells from different mammalian species as well as cells at different differentiation stages, from pluripotent to differentiated cells [53, 54, 61, 64]. Interestingly, although in somatic cells constitutive heterochromatin around centromeres was shown to replicate late and to follow replication of the facultative heterochromatin, in mouse pluripotent cells this order was reversed. (Epi)genetic manipulation experiments combined with replication progression studies, as just described, uncovered the histone acetylation level of chromatin as one of the most relevant epigenetic mechanisms regulating ordered progression of genome replication. While these approaches do not often provide DNA sequence information, they yield replication information in single cells in 3D and with high time resolution. Furthermore, until very recently genome-wide sequence analysis did not take into consideration repeat elements, including interspersed and tandem repeats, which make up about 50% and 10% of the genome in mammals, respectively. This type of information is easily accessible from microscopy-based approaches but only recently available for the human genome [75, 76] and, in part, for the mouse genome [77]. Although replication of specific chromosomes is not easily analyzed with microscopy-based methods, the availability of DNA paint probes for human and mouse chromosomes [78] permitted addressing the replication timing of some chromosomes, such as the inactive versus active X chromosome in somatic female mammalian cells as well as the Y chromosome. The combination with epigenetic markers and FISH probes indicated that the inactive X and Y chromosomes replicate in a short and synchronous manner with most origins firing simultaneously in mid S phase for the inactive X [79] and late S phase for the Y chromosome [61]. For the inactive X chromosome, histone acetylation [79] and also histone variant macroH2A1 were found to play a role in this synchronous mode of replication [80]. Interestingly, this mode of synchronous replication is reminiscent of the early embryonic stages in organisms such as *Drosophila melanogaster* and *Xenopus laevis* before embryonic transcription starts, which has analogies with these mostly transcriptionally silenced chromosomes.

While microscopy-based approaches revealed the spatiotemporal choreography of RFi within the nucleus, they provided limited information about which genomic sequences were being replicated and when. Conversely, the genome-wide approaches described in the following section sacrificed spatial resolution within the cell in favor of sequence-level information. The power of the field today lies in combining both.

**Figure 3.**
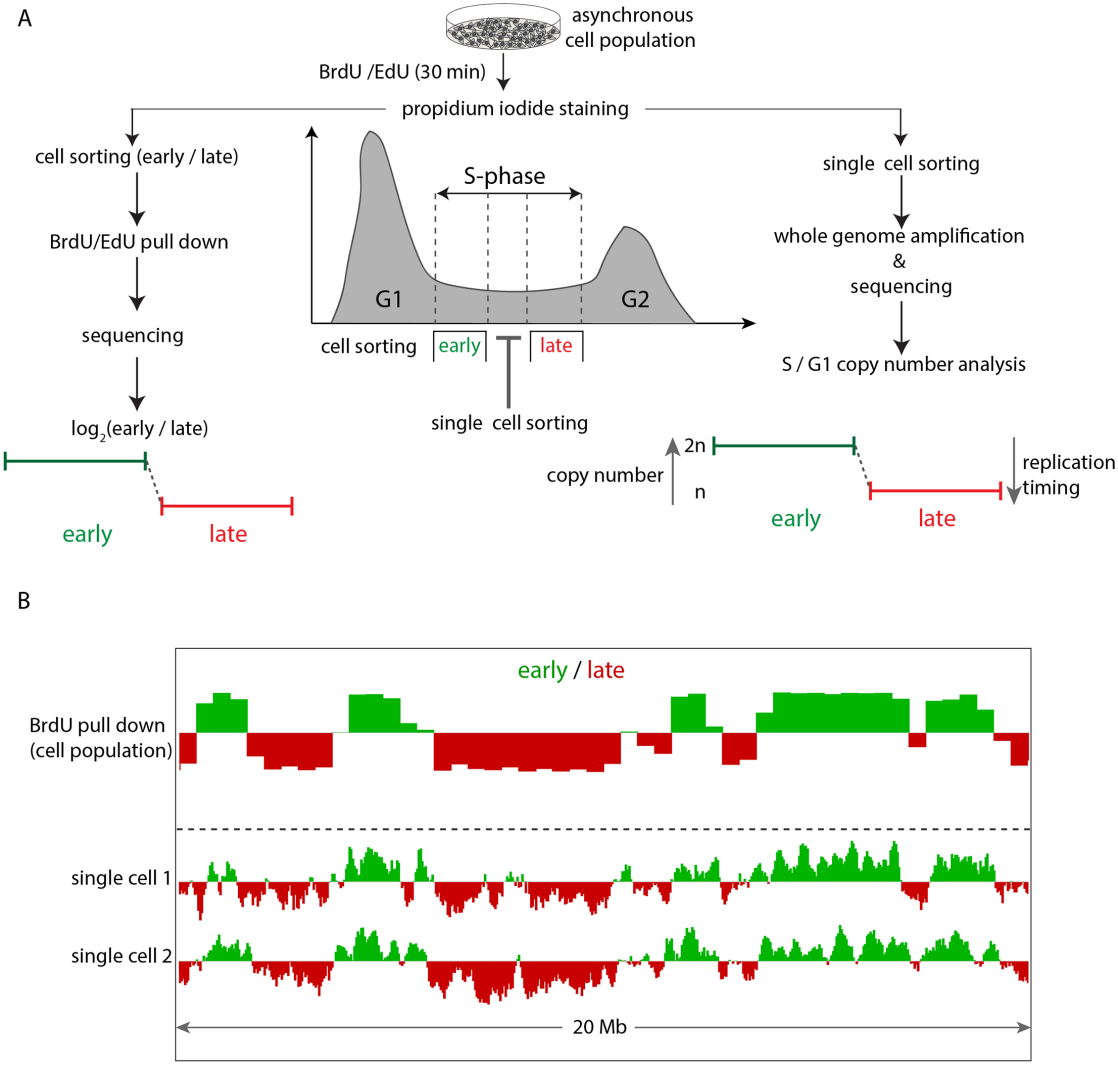
Genome-wide replication timing (repli-seq) analysis. (**A**) The scheme shows approaches to map the replication timing in cell populations or individual single cells. The representative plot depicts the DNA content histogram of an asynchronous cell population. For BrdU/EdU pull-down, the cell population with a pulse of 30 min is sorted into early or late S phase based on DNA content. Nascent DNA is pulled down and hybridized to microarrays or sequenced. The replication time is inferred based on the signal intensity of the hybridized probes or sequencing reads [log2(early/late)]. For mapping replication timing in individual cells, the single cells gated from the middle of the S phase are subjected to whole-genome amplification, sequencing, and copy number normalization to G1 cells. Both methods reveal replication timing in cells. (**B**) Replication timing profiling by repli-seq methods shows genome-wide stability at the population level and cell-to-cell stochastic variation in early and late replicating chromatin domains. The repli-seq data plotted were accessed from GEO: GSE108556 [91].

With the human/mouse genome project and advances in microarray technology, a broad view of the conserved replication timing was revealed [67, 81–86]. Table 3 summarizes genome-wide replication origin mapping as well as replication timing studies and Fig. 3 provides a simplified overview of the replication timing mapping methodology. A comprehensive review of replication mapping techniques can be found in Hu *et al*. and Hyrien *et al*. [2, 3]. These studies unraveled that gene-rich chromosomes (like human chromosomes 22, 19, and 17) replicate earlier in the S phase, while others (e.g. human chromosomes 18, 21, and Y) replicate later. Furthermore, while the GC- and Alu-rich regions were replicated earlier, the LINE-rich regions were replicated later. With the advance of next-generation sequencing, a comprehensive view of the replication timing was obtained by sequencing the nascent DNA [87]. It showed the genome duplicates in megabase-sized replication domains, where the stretch of the DNA shares the same replication timing [88]. The outcome of these genome sequencing data agreed with earlier microscopic RFi analysis in cells quantifying the average amount of DNA per replication focus [86]. In addition, these studies also characterized the constant and plastic replication domains, where the replication timing of the constant domains remains the same for different cell types, and the plastic domains change with developmental stage or cell type. However, these studies, being based on cell populations, did not reveal the cell-to-cell variation or stability in replication timing until advances in single-cell genome amplification methods and copy number analysis paved the way to investigate these replication domains in individual cells [89]. By collecting the cells in the middle of S phase based on the DNA content and performing the copy number gain due to genome duplication, the replication timing in single cells was analyzed (Fig. 3) [90, 91]. The outcome uncovered the genome-wide stability of these replication domains among cells, with a certain degree of stochastic variation from cell to cell, especially in the late S phase. As mentioned above, these studies suffered from the lack of a complete sequence from telomere to telomere (T2T) of human (and mouse) chromosomes, and did not address replication of repeat elements, which constitute a large portion of mammalian genomes. The recent compilation of the T2T sequence of the human genome [75, 76] has allowed remapping of the original replication sequencing data to create a more comprehensive analysis [92]. Altogether, we now have a compendium of replication timing data from a variety of species and cell types at a very high sequence resolution level (resources @ 4dnucleome.org and @ encodeproject.org). Remapping of these data to the full genome sequence assemblies becoming available will be required to yield complete genome sequence views of replication timing and its changes during development and disease. These sequencing data confirm and extend the notion that euchromatin replicates in general earlier than heterochromatin, which had been already established by microscopical analyses dating as far back as the 1960s with the work of Lima-de-Faria among others (see Lima-de-Faria *et al*. [30] and references therein). This notion simultaneously raises the question of how genome replication spreads from early to late firing origins.

**Table 3.** Summary of genome-wide (origin) replication mapping studies in mammalian cells

Year	Landmark	Author
1985	Isolation and characterization of early replicating sequences in monkey	Kaufmann *et al*. [93]
2001	Developmental regulation of human ꞵ-globin replication timing	Simon *et al*. [94]
2004/2008	Replication timing of human and mouse genome using microarray and effect of developmental stage on replication timing	Woodfine *et al*.; Farkash-Amar *et al*.; Hiratani *et al*. [83, 84, 88]
2008/2009	Genome-wide SNS-seq in human and mouse cells	Cadoret *et al*.; Sequeira-Mendes *et al*. [95, 96]
2010	Genome-wide replication timing using BrdU pull-down and next-generation sequencing	Hansen *et al*. [87]
2011/2013	Purification and analysis of replication bubbles to infer replication origins	Mesner *et al*.; Mesner *et al*. [97, 98]
2012/2019	Correlation of G-rich motifs with replication origins and functional demonstration of origin activity of G-quadruplex regions	Cayrou *et al*.; Besnard *et al*.; Prorok *et al*. [99–101]
2013	Genome-wide mapping of human DNA replication origins shows transcription regulating origin selection and replication timing	Dellino *et al*. [102]
2013/2018/2019	Genome-wide stability and stochastic genome replication program inferred from single-cell repli-seq	Van der Aa *et al*.; Dileep and Gilbert; Takahashi *et al*. [90, 91, 103]
2016	Inferring replication fork directionality using OK-seq	Petryk *et al*. [104]
2018/2020/2024	Replication program of repetitive elements inferred from repli-FISH	Natale *et al*.; Rausch *et al*. Pradhan *et al*. [61, 64, 67]
2020	Temporal progression of genome replication inferred from high-resolution repli-seq	Zhao *et al*. [105]
2021	Genome-wide replication program using optical mapping in nanochannels	Wang *et al*. [22]
2022	Remapping of human replication timing data to the T2T sequence	Massey and Koren [92]
2022	Genome-wide origin mapping with Ini-seq 2 in human cells	Guilbaud *et al*. [106]
2024	Emergence of the replication program in early development	Nakatani *et al*.; Takahashi *et al*.; Halliwell *et al*. [107–109]
2024/2025/2026	Nanopore-sequencing based replication mapping	Carrington *et al*.; Jones *et al*.; Rojat *et al*. [110–112]

SNS: Short nascent strand, OK: Okazaki fragment, FISH: fluorescence *in situ* hybridization

In parallel, advancements in characterizing the replication origins in mammalian cells also happened using microarrays and next-generation sequencing. In *Saccharomyces cerevisiae*, origin selection is guided by the binding of ORC to well-defined DNA sequences near ARS elements [113]. However, unlike in yeast, the ORC from higher eukaryotes exhibits no sequence specificity *in vitro* [114] and metazoan replication origins have not been associated with a single conserved consensus sequence, although enrichment in G-rich elements and G-quadruplex motifs has been reported at many origins [3, 99, 100]. Development in tools to map these origins, such as SNS isolation in mammalian cells (Fig. 2B), supported these findings [95]. The sequencing of the Okazaki fragments (OK-seq) (Fig. 2B) revealed the initiation zones (located primarily within non-transcribed, broad up to 150 kb zones that often about transcribed genes), the direction of the replication forks, and the termination sites. It should be noted that the OK-seq method maps Okazaki fragment strand transitions and, therefore, replication fork directionality across broad initiation zones, typically detecting 5000–10 000 such zones rather than individual origins. In regions where multiple potential origins are closely spaced, signals from different cells are averaged, smoothing sharp transitions into broad zones and underestimating the total number of origins [104]. Further investigations related to the origin positioning with respect to the transcription start site and the secondary structure of the DNA, with G-quadruplex motifs emerging as a recurrent feature at a subset of origins [26, 96, 97]. A comprehensive characterization of human replication origins, including sequence consensus, correlations with topologically associated domains (TADs), and deregulation in immortalized cells, was also reported [115]. Nevertheless, a universally shared sequence determining metazoan origins remains elusive (reviewed in Hyrien *et al*. [3]). Isolating a sufficient amount of the Okazaki fragments or the SNS has been challenging, making it difficult to infer the origin feature on a single-molecule scale, as seen in the combed fibers and cell-to-cell variation [116]. Recent advancements in next-generation long-read sequencing (e.g. nanopore sequencing) and detection of the incorporated nucleotide analogs (BrdU or EdU) can reveal the single-molecule information and more features about these origins, especially in the highly repetitive genomic regions [110–112, 117]. Nonetheless, the origin of mammalian DNA replication is still an active and challenging research topic, and a consensus has yet to be reached about its features and specification.

One aspect that remains largely unaddressed in metazoan origin mapping studies is origin selection and firing within repetitive sequences, which constitute the majority of mammalian genomes. Genome-wide sequencing approaches are inherently limited in this regard, as short reads cannot be uniquely mapped to repetitive elements, effectively rendering these regions invisible to SNS-seq, OK-seq, and related methods. Notable exceptions include studies showing that transcription constrains replication initiation to intergenic sequences at rDNA loci during embryonic development in *Xenopus* [118], but systematic origin mapping within satellite DNA, transposable elements, and other repetitive sequences in mammalian cells remains essentially uncharted. Long-read nanopore sequencing, combined with T2T genome assemblies, now offers a realistic path toward addressing this gap, and cellular approaches such as Repli-FISH have already provided initial insights into the replication timing of these elements.

### From the replication foci back to replication forks

The above studies of replication timing and origins revealed that while the replication timing remains globally conserved, the replication origins are plastic. This suggested that the temporal order of firing the initiation sites, rather than the sites themselves, maintains the replication program, yet its mechanism and significance are unknown. Overall, the broader understanding of the replication program narrows down to the understanding of the principles behind the spreading of DNA replication throughout the chromosomes. The microscopic dissection of the spatiotemporal replication program *in vivo* has bridged many such gaps in our understanding. This approach already showed the conserved spatiotemporal propagation of RFi in S-phase stages [119–121]. Live cell imaging of the GFP-tagged replication protein PCNA showed the dynamic nature of the RFi that assemble and disassemble (rather than moving, merging, or dividing), creating differential spatial patterns in respective S-phase stages [54]. High-time-resolution microscopy coupled with fluorescence recovery after photobleaching (FRAP) revealed the stable association of replisome machinery components with the RFi. This led to the proposal that replication progresses in a domino-like next-in-line model [55]. Quantitative analysis of human genome replication integrating DNA combing with massive sequencing of newly replicated DNA showed that origins are activated synchronously in clustered regions of shared replication timing. However, they are activated gradually in temporal transition zones, with the rate of origin firing increasing as replication progresses [122]. In this context, origin interference occurs when the distance between the two origins is low, usually <100 kb, and an average of ~40 kb [123]. Based on the existing observations, the “domino model” of replication progression materialized: stochastic activation of the first origin clusters leads to a chain reaction of sequential activation of later origin clusters depending on the relative spatial distribution of the genome within the nucleus. Yet the origin interference kicks in within a short distance, usually when the next origin is present in the same chromatin loop [124]. The model could capture the spatiotemporal replication progression and replication timing as observed in microscopic and repli-seq analysis. Alternative models in fission yeast [65], including purely stochastic origin firing without spatial coupling, have also been proposed.

The original attempts to label and quantify replicating foci in mammalian cells (Table 2) detected a mere few hundreds of actively replicating foci, occurring in a sequential order, distributed across different subnuclear locations throughout the S phase [50, 125, 126]. Together with fork speed and replicon size data obtained primarily from DNA fiber analysis, these numbers could not explain and account for how mammalian chromosomes get duplicated during the S phase. The emergence of digital imaging in the mid 1990s and computational image analysis led to better quantification of RFi in 3D fluorescence microscopy images, yielding around a thousand foci. The later development of superresolution light microscopy (reviewed in Schermelleh *et al*. [127]), using either nucleotide pulse labeling as well as antibodies to replisome components and/or fluorescence fusions thereof, yielded three to five thousands of replication sites (dependent on the ploidy of the cell) to be mapped at any given time of the S phase each about 120 nm diameter in size [58, 59, 61, 64]. Integrating data from DNA fiber replication analysis with measurements of the duration of S phase and the number of nanoRFi at any given S-phase stage led to the suggestion of clusters of replicons (aka, nanoRFi) as the base unit of chromosome replication [42, 86, 121] i.e. a segment of DNA synthesized from a single origin. Such units are often fired together in time and space, giving rise to the observed clusters of nanoRFi seen in microscopical analysis and the clusters of replicons seen in DNA fiber analysis. While the replication timing and nanoRFi numbers between different studies are reproducible, origin mapping studies are highly resolutive but less concordant between data sets. Independent studies using λ-SNS, BrdU-SNS, or replication bubble-containing EcoRI fragments showed only 11%–35% pairwise origins matching. Sequenced λ-SNS from human HeLa and three other cell lines showed peak clustering into zones. However, the complete sequencing set of λ-SNS in HeLa only overlapped 51% with other studies. In addition, bubbles and SNS showed different conservation between cell lines, with λ-SNS being more conserved, overlapping 65%–84% [116]. Hence, the number of origins derived from DNA fiber replication analysis and corresponding numbers of nanoRFi provide a very much needed cross-validation and a rationale for thresholding the replication origin sequencing data.

The sizes of replicons and the mechanistic explanation of their clustering and replication timing have been proposed in earlier studies. Already in 1965, Lima-de-Faria *et al*. observed that different types of heterochromatin in male and female cells, nucleolus-associated and scattered heterochromatin, synthesized their DNA at different periods from euchromatin [128]. In 1968, Lima-de-Faria and Jaworska wrote about chromosome replication: “It seems to be a rule of chromosome replication that heterochromatin synthesizes its DNA at a later stage than the rest of the chromosome” [30], a rule still valid in the present. By the mid 1970s, a close relationship was observed between replication timing, chromatin structure, and transcriptional activity. A study showed that late-replicating DNA coincided with AT-rich, Giemsa-dark, G-bands on metaphase chromosomes while early-replicating DNA coincided with GC-rich, R-bands with high transcriptional activity [129]. As it was shown later, early and late replicating sequences occupy different subnuclear compartments [130]. However, even nowadays, replication timing is often discussed superficially in terms of its relationship with transcriptional activity and chromatin structure. Little is known about the real mechanistic and causal relationship between transcription and replication.

In 1980, the DNA halo technique was first applied, allowing visualization of a fluorescent halo made of DNA loops extruded from the nuclear scaffold [131]. They measured loops with an average size of 90 kb in mouse cells, identifying a relationship between DNA loops and replication, which was confirmed and extended by Buongiorno-Nardelli *et al*. in 1982 [132]. Buongiorno-Nardelli and colleagues proposed that the maximum halo radius or loop size is directly proportional to the average replicon length by comparing the loop size estimated with the halo method and the replicon size known from fiber autoradiography. They speculated that a replicon might consists of two adjacent loops, having origins and terminations at the anchors of the loops [132]. Therefore, replicon size is determined by the spacing between active origins, which is correlated with the length of chromatin loops [132]. The average size of these chromatin loops ranges from 60 to 185 kb, depending on the study (reviewed in Mamberti *et al*. [133]). Therefore, these substructures, dependent on structural proteins like cohesin [134] or histone variants enrichment [80], regulate the spatial organization of origin clusters. On the other hand, a strict correlation between replication speed during a given S phase and the size of chromatin loops in the next G1 phase has been shown, suggesting a loop-dependent mechanism of origin programming [135].

**Figure 4.**
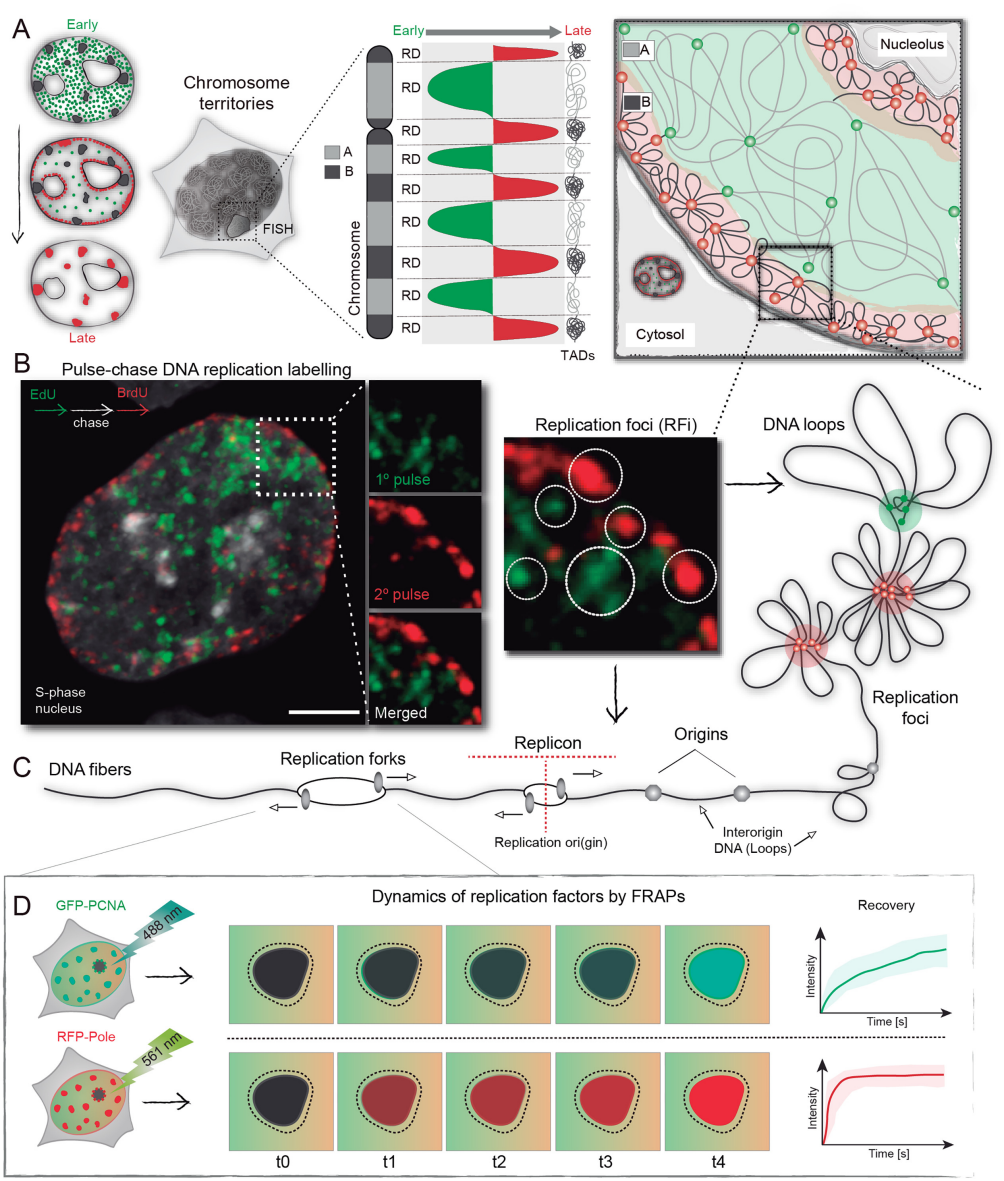
Correlation of genome-wide replication and chromosome conformation, from foci to forks. (A–C) Graphical representation of hierarchical DNA folding through chromatin organization and the spatiotemporal regulation of DNA replication from the genome to individual replisome/replicon. (**A**) On the top left, replication timing is reflected in genome architecture, displaying a specific subnuclear distribution or S-phase patterns, shown as a diagram: early (green foci), mid, and late (red foci). The latter illustrates the spatiotemporal procession of DNA replication, determined by chromosome conformation. Selecting a chromosome territory as an example (scheme of the interphase nucleus) and zooming in on it, the peak graph illustrates the relationship between replication timing and chromatin structure: early (green) versus late (red) replicating domains (RD), ~1–5 Mb regions. These RD are chromatin segments that coordinately switch replication timing during cell fate change. RDs share the properties and approximate boundaries of a subset of TADs and align with TADs that are at compartment boundaries. Early replication timing corresponds to A compartments, but late replication timing corresponds to B compartments (right top). Schematic magnification of a region in the nucleus shows the model for genome compartmentalization into early and late replicating chromatin regions and their subnuclear position. Green and red foci correspond with the RDs in the graph to the left. (**B**) Zooming in on the replicating A/B compartments, the scheme in the panel on the right represents DNA loops as subcompartment structures regulating interorigin distances and origins clustering. These origin clusters are visualized as RFi in microscopy studies: panel (B)-left shows an S-phase nucleus in which nascent DNA has been labeled with modified nucleotides (EdU and BrdU). In these studies, the spatiotemporal progression of replication can be analyzed by pulse-chase-pulse DNA replication labeling, reflecting the model’s accuracy. Magnification shows the RFi for the first pulse (green-early A compartment) and the second pulse after chase (red-late B compartment) in relation to the nuclear periphery. (**C**) These foci can be visualized “unfolded” as DNA fibers, where they can be related to individual replication forks and single replicons. (**D**) Schematic representation of FRAP experiments and their potential to study the association dynamics of replication-associated factors in live cells. Double FRAP performed in the same S-phase cell can be used to study the binding of GFP-PCNA and RFP-Pole, obtaining fluorescence recovery curves. Analysis of FRAP provides information on the exchange rate and when and where these factors are reloaded.

The development of approaches to study genome-scale analysis of replication timing by next-generation sequencing (Fig. 3) provided an exponential increase in the possibilities of answering these mechanistic questions, mapping replication timing domains genome-wide and, more recently, in single mammalian cells [136], and correlating replication timing domains with A/B compartments detected by Hi-C. These studies led to the finding that replication timing is regulated at the level of large chromosomal domains. A domain of early or late replicating sequences comprises multiple TADs, with replication timing boundaries matching TADs boundaries (Fig. 4A) [137]. Therefore, it was proposed that TADs could be regulatory units of replication timing. They are also structural units of chromosomes, reflecting chromatin states and different subnuclear positioning. However, the precise relationship between TADs and the 1-Mb chromatin domains (RFi) is not fully established, although their size is similar. Important limitations of the genomic approach lie in underrepresenting the highly abundant repetitive genomic elements replicating during late S phase, such as tandem repeats in heterochromatic regions. The combination of genomic population-level data with single-cell immunofluorescence replication and FISH (Repli-FISH) of repetitive DNA elements was able to address the replication timing of Alu, LINE-1, and satellite repetitive elements [67]. PacBio and nanopore sequencing, as long-read sequencing technologies, offer unique advantages for studying the replication of repeat elements, which are often underrepresented (filtered out) in short-read sequencing data. In addition, both PacBio and nanopore sequencing can detect DNA modifications like 5-methylcytosine without chemical conversion steps [138–140], giving the possibility to correlate the replication features of the sequences with epigenetic marks. Despite its advantages, long-read sequencing has limitations, including higher costs, lower throughput compared to short-read technologies, and the need for specialized protocols. For mapping origins and their distances, long-read sequencing with an average read length of 100 000 bp remains limiting as interorigin distances are typically over this size range. In this context, many more methods have been developed for nanopore sequencing: DNAscent v2, designed to detect replication forks in nanopore sequencing data using deep learning [141], FORK-seq to map replication of single DNA molecules at 200-nucleotide resolution [142], NanoForkSpeed combining nanopore sequencing with pulse-labeling [143], and MATAC-seq to study DNA replication origin efficiency [144], among others.

Studies using fluorescence as well as electron microscopy analysis of cell pulse labeled with modified nucleotides led to the concept of replication factories, which provided a different mechanistic explanation for how replicon units clustered [33, 34, 36]. This “replication factory” model described replication as occurring within defined fixed subnuclear structures, where the replication machinery concentrated, termed replication factories. These factories were visible even when digesting away >90% of the cellular DNA. The model proposed that during replication, DNA from adjacent chromatin must be translocated to these active sites to be replicated and then extruded out of the synthetic factories. Whether the replication factories were assembled prior to the S phase or only during S phase was unclear. The model further proposed that the factories of synthetic machineries were attached to an insoluble nuclear skeleton/matrix likely composed of intermediate filament proteins. As it so happens, this concept of replication factories is most often referred to in a simplistic and incorrect manner that does not reflect its basic proposal that replication machineries cluster together in the absence of DNA but are bound to an insoluble nucleoskeleton. The lack of biochemical evidence on the existence and nature of the postulated nucleoskeleton/matrix spurred a lot of controversy in the field. Furthermore, parallel biochemical *in vitro* work on the composition of the (eukaryotic) replisome and the respective enzymatic activities projected a notion of soluble replication factors building multiprotein complexes (reviewed in Yao *et al*. [145]). This counteracted the replication factory model in mammalian cells, where replisome factories were formed as insoluble structures. The “insoluble solution” to replication in cells had, though, the advantage of providing easy coordination of enzymatic activities in specific sites within the cell nucleus (reviewed in Leonhardt *et al*. [146]). Subsequent analysis of replisome components in living cells by engineering replisome components as fusions with fluorescent proteins revealed that some replisome factors bind very dynamically within active RFi, whereas others remained mostly stably bound to the replicating DNA [55, 56, 147]. Whether the stably bound replisome components require the nucleoskeleton is so far not definitely answered, but the absence of specification of its molecular composition makes it difficult to uphold. Nonetheless, as was shown before for prokaryotic systems, the eukaryotic replisome can be reconstructed *in vitro* in solution and recent work has demonstrated that even a functional human replisome can be reconstituted [148]. New developments using AI-based modeling of interactions with AlphaFold have paved the way for an *in silico* detailed analysis of the replisome organization and for the discovery of new replisome components [149].

Thanks to technological and computational advances, the gap between genome-wide studies and microscopic data on chromatin structure and RFi is becoming increasingly close. Early and late replication domains detected by repli-seq correlate with A (active) and B (inactive) compartments defined by chromosome conformation capture studies (Hi-C), consistent with the spatial pattern of early and late RFi revealed by microscopy (Fig. 4A and B). The recent feasibility of single-cell replication analysis (scRepli-seq) has allowed genome-wide replication timing mapping of mouse embryos, showing that replication timing definition coincides with the strengthening of A and B compartments [107]. Nonetheless, single-molecule and super-resolution microscopy studies in mouse embryonic stem cells identified replication timing switches compared with differentiated cells [61]. scRepli-seq, together with genome-wide approaches like pulse-chase-pulse experiments, is a powerful tool to understand the relationship between replication timing and 3D genome organization at the single-cell level, and how they are regulated by chromatin structure, transcription-associated events, imprinting, and/or epigenetic marks. Table 4 summarizes studies relating chromosomal structure to DNA replication regulation.

**Table 4.** Landmarks in structural chromatin units and regulation of DNA replication

Year	Landmark and structure	Author
1958	Confirmation of the semiconservative DNA replication model	Meselson and Stahl [7]
1965	Late replication of autosomal heterochromatin	Lima-de-Faria *et al*. [128]
1967	Patterns of chromosome replication by H^3^-thymidine labeling and karyotype analysis	Lima-de-Faria *et al*. [150]
1977/1983	Histone-depleted metaphase chromosomes—loops	Paulson and Laemmli; Earnshaw and Laemmli [151, 152]
1980	DNA halo technique—loops and DNA replication	Vogelstein, Pardoll, and Coffey [131]
1981	RFi visualized using premature chromosome condensation	Lau and Arrighi [153]
1982	Relationship between average loop length (DNA loops) and replicon size in different animal and plant species	Buongiorno-Nardelli *et al*. [132]
1998	Stable replicon clusters as units of chromosome structure	Jackson and Pombo [121]
1999/2001	The link between spatial position and replication timing of chromosomal domains established during G1	Dimitrova and Gilbert; Li *et al*. [154, 155]
2004	Stable chromosomal units control the spatial and temporal organization of DNA replication	Sadoni *et al*. [42]
2009/2012/2014	Hi-C^a^ methodology identifies megadomains and A/B compartments, TADs^b^, and loop domains	Lieberman-Aiden; Dixon *et al*.; Rao *et al*. [156–158]
2012	Late replication of constitutive heterochromatin requires histone hypoacetylation	Casas-Delucchi *et al*. [159]
2018/2021	DNA replication dynamics of repeat elements; Alu versus L1^c^ retrotransposons enriched in A and B compartments, respectively	Natale, Scholl, Rapp *et al*.; Lu *et al*. [67, 160]
2018/2019	First genome-wide scRepli-seq showing differences between active/inactive compartments and haplotype resolution	Dileep and Gilbert;Takahashi, Miura *et al*. [90, 91]
2018	scRepli-seq shows allele-specific control of replication timing and genome organization during development	Rivera-Mulia [161]
2019/2022	Single-cell replication profiling shows the developmental dynamics of chromosome organization mapping replication timing	Miura *et al*. Bartlett *et al*. [162, 163]
2020	Repli-seq method defines the temporal progression of initiation, elongation, and termination of replication in mammalian cells	Zhao *et al*. [105]
2024	Regulation of replication synchrony in the inactive X chromosome	Arroyo *et al*. [80]
2024	Establishment of replication timing during development related to the formation of A/B compartments	Nakatani *et al*. [107]
2025	Non-coding RNAs as critical regulators of replication timing in constitutive heterochromatin	Pradhan et el. [164]

a High‐throughput chromosome conformation capture.

b Topologically associated domains.

c LINE 1.

In addition to mapping replication units and discrete origins within chromosomes, equally important is to ascertain the spatial and temporal dynamics of the components of the replisome. To this purpose, evolutionary conservation of factors as well as methods to pull down nascent DNA-associated factors have been very powerful for the discovery of new regulatory networks. To further comprehend how replication factors come together to form synthetic units throughout the S phase, kinetic measurements are required. This involves engineering to allow for their visualization in live cells. Furthermore, and in contrast to static and structural approaches to reconstitute the replisome [165], FRAP-type experiments are a powerful tool to study the replication dynamics in live cells (Fig. 4D). Photobleaching analyses first showed the different recovery kinetics of PCNA and RPA in replicating cells and, more importantly, that *de novo* assembly of replication sites occurs close to earlier ones following the domino effect [55]. Beyond the detailed study of replication factors and their association kinetics, this experimental approach could be implemented under replicative stress conditions and to investigate the effect of mutations. Although cryo-electron microscopy can resolve complex structures with a high degree of resolution, including contacts, positions, and binding between multiple replisome components, it lacks the functionality to study these components in their changing cellular environment. Thus, live cell microscopy approaches, such as single-molecule tracking/counting and photobleaching of replication-associated factors, have the potential to uncover the intricate dynamic network of actions of these complexes. Finally, the increasing number of factors involved in synthesizing as well as regulating the synthesis of chromosomes demands resolving their stoichiometry and spatio–temporal relationships within each fork in the cell. Importantly, merging all these data together will uniquely provide us with a mechanistic view of the replisome and its associated replication fork in action.

### Conclusions and outlook

Decades of research confirm that DNA replication is an intrinsically ordered process, physically coupled to the 3D genome architecture. Though most replication factors have been cataloged and structurally resolved, the field must now pivot from descriptive mapping to a mechanistic understanding of the dynamic forces governing this program. This transition is essential to address the central paradox of mammalian replication: the strict conservation of replication timing persists despite the highly plastic nature of origin selection, demanding that future inquiry focus on defining the non-sequence-based regulatory layer that coordinates factor activity at the cellular level for efficient and precise genome duplication. Crucially, the precise mechanistic basis for the domino-like spatial propagation of replication initiation remains elusive, necessitating investigation into whether it is driven by a diffusible signal, a mechanical consequence of loop extrusion, or a localized chromatin state transition.

It is inadequate to propose that this regulation is governed solely by static epigenetic marks or generic spatial position. Instead, the execution and fidelity of the replication program are likely dictated by a mechanism wherein molecular regulators impose biophysical constraints on the chromatin template. Future research must therefore focus on establishing the causal and reciprocal relationship between specific molecular factors and the resulting physical properties of chromatin. We must define how transient molecular events, such as the binding kinetics of replication timing regulators, the precise localization of heterochromatin marks, or the influence of specific non-coding RNAs, can create predictable physical constraints on the chromatin fiber, such as changes in local mobility and compaction.

To achieve this critical insight, the field demands a methodological synthesis. While the field has significantly benefited from *in vitro* reconstitution and structural biology in cataloging the essential replisome components, the path forward requires a necessary pivot: transitioning from these isolated biochemical systems to prioritizing the real-time kinetic interrogation of the replisome *in vivo*. This shift is crucial to determining the functional consequences of molecular regulation. Live cell microscopy with fluorescent labeling of synthesized DNA and proteins offers the possibility to probe the dynamics of these large molecular assemblies and investigate how they behave during initiation/termination, respond to transcriptional clashes, DNA damage, and cellular stress to maintain genome integrity over countless replication cycles, even under challenging environmental conditions or age-related reduced cellular fitness. Integrating high-resolution kinetic measurements (like FRAP and single-particle tracking) that capture the dynamics of replisome components *in vivo*, with multi-omic single-cell approaches that simultaneously map replication status, epigenetic profiles, and 3D nuclear topology, will be necessary. This will overcome the averaging effect of population studies and provide the resolution needed to link molecular biochemistry to the robust, conserved replication program observed at the cellular level. The next mechanistic frontier hinges upon successfully demonstrating how molecular mechanisms exert control through the physical manipulation of the chromatin template, ensuring global genomic stability across cell generations. In the future, better methods to integrate data from all the approaches discussed will be of paramount importance for a comprehensive understanding of how genomes are maintained.

Several key questions remain unresolved. What determines which of the thousands of licensed origins actually fires in a given cell cycle? What is the mechanistic basis for the domino-like spatial propagation of replication initiation—is it a diffusible signal, a mechanical consequence of loop extrusion, or a chromatin state transition? How does the replication program change during aging and disease, and can replication timing serve as an epigenetic clock analogous to DNA methylation-based clocks? Answering these questions will require integrating the complementary strengths of the approaches reviewed here into unified multi-modal experimental frameworks.

## Data Availability

No new data were generated or analyzed in support of this research.
